# Association between circulating 25-hydroxyvitamin D and incident type 2 diabetes: a mendelian randomisation study

**DOI:** 10.1016/S2213-8587(14)70184-6

**Published:** 2015-01

**Authors:** Zheng Ye, Stephen J Sharp, Stephen Burgess, Robert A Scott, Fumiaki Imamura, InterAct Consortium, Claudia Langenberg, Nicholas J Wareham, Nita G Forouhi

**Affiliations:** a MRC Epidemiology Unit, University of Cambridge School of Clinical Medicine, Institute of Metabolic Science, Cambridge, UK; b Department of Public Health and Primary Care, University of Cambridge, Cambridge, UK

## Abstract

**Background:**

Low circulating concentrations of 25-hydroxyvitamin D (25[OH]D), a marker of vitamin D status, are associated with an increased risk of type 2 diabetes, but whether this association is causal remains unclear. We aimed to estimate the unconfounded, causal association between 25(OH)D concentration and risk of type 2 diabetes using a mendelian randomisation approach.

**Methods:**

Using several data sources from populations of European descent, including type 2 diabetes cases and non-cases, we did a mendelian randomisation analysis using single nucleotide polymorphisms (SNPs) within or near four genes related to 25(OH)D synthesis and metabolism: *DHCR7* (related to vitamin D synthesis), *CYP2R1* (hepatic 25-hydroxylation), *DBP* (also known as *GC*; transport), and *CYP24A1* (catabolism). We assessed each SNP for an association with circulating 25(OH)D concentration (5449 non-cases; two studies), risk of type 2 diabetes (28 144 cases, 76 344 non-cases; five studies), and glycaemic traits (concentrations of fasting glucose, 2-h glucose, fasting insulin, and HbA_1c_; 46 368 non-cases; study consortium). We combined these associations in a likelihood-based mendelian randomisation analysis to estimate the causal association of 25(OH)D concentration with type 2 diabetes and the glycaemic traits, and compared them with that from a meta-analysis of data from observational studies (8492 cases, 89 698 non-cases; 22 studies) that assessed the association between 25(OH)D concentration and type 2 diabetes.

**Findings:**

All four SNPs were associated with 25(OH)D concentrations (p<10^−6^). The mendelian randomisation-derived unconfounded odds ratio for type 2 diabetes was 1·01 (95% CI 0·75–1·36; p=0·94) per 25·0 nmol/L (1 SD) lower 25(OH)D concentration. The corresponding (potentially confounded) relative risk from the meta-analysis of data from observational studies was 1·21 (1·16–1·27; p=7·3 × 10^−19^). The mendelian randomisation-derived estimates for glycaemic traits were not significant (p>0·25).

**Interpretation:**

The association between 25(OH)D concentration and type 2 diabetes is unlikely to be causal. Efforts to increase 25(OH)D concentrations might not reduce the risk of type 2 diabetes as would be expected on the basis of observational evidence. These findings warrant further investigations to identify causal factors that might increase 25(OH)D concentration and also reduce the risk of type 2 diabetes.

**Funding:**

UK Medical Research Council Epidemiology Unit and European Union Sixth Framework Programme.

## Introduction

Vitamin D is a steroid hormone that has a crucial role in the modulation of bone homeostasis. It has been described as a wonder vitamin because of its possible benefits related to diverse health outcomes including bone disease, coronary heart disease, and type 2 diabetes.^1^, ^2^, ^3^ 25-hydroxyvitamin D (25[OH]D) is a circulating metabolite used as a clinical indicator of vitamin D status. Results from prospective epidemiological studies have shown that low circulating 25(OH)D concentrations are associated with an increased risk of developing type 2 diabetes.^3^, ^4^ However, whether or not this association is causal is unknown;^3^ it might be the result of residual confounding, which is plausible in observational studies of incident type 2 diabetes. Measurements of confounders (eg, physical activity) are susceptible to errors and are not adequately controlled for in epidemiological analyses.^5^ Although results from clinical trials^6^, ^7^ have shown no effect of vitamin D supplementation on the incidence of type 2 diabetes, these findings require cautious interpretation because of issues with doses, combination treatment with calcium, compliance, and generalisability.^3^

Studies of genetic variants (single nucleotide polymorphisms, SNPs) that specifically affect 25(OH)D concentration can provide another route to draw causal inference. Investigators of a genome-wide association study^8^ identified SNPs within or near four genes significantly related to 25(OH)D concentrations: *DHCR7* (encoding 7-dehydrocholesterol reductase; involved in 25[OH]D synthesis in the skin) and *CYP2R1* (encoding cytochrome P450, family 2, subfamily R, polypeptide 1; involved in hepatic 25-hydroxylation), both representing 25(OH)D synthesis; and *DBP* (also known as *GC*; encoding vitamin D-binding protein; involved in 25[OH]D transport) and *CYP24A1*; (encoding cytochrome P450, family 24, subfamily A, polypeptide 1; involved in 25[OH]D catabolism), both representing vitamin D metabolism. SNPs within or near these genes have been investigated as instruments to predict 25(OH)D concentrations independent of potential confounders and to study causal associations of 25(OH)D concentrations with health outcomes.^2^, ^9^, ^10^, ^11^, ^12^ Using a mendelian randomisation approach, investigators of previous studies have examined causal associations of 25(OH)D with risk of type 2 diabetes, but these studies were unable to confirm or refute causality because of small sample sizes, few included SNPs, or the absence of validation (ie, through assessment of intermediate risk factors including glycaemic traits).^9^, ^10^, ^11^ Larger studies are needed to assess more precisely the potential causal association between 25(OH)D concentrations and risk of type 2 diabetes. If causality does exist, interventions such as sunlight exposure or increased vitamin D intake (diet or supplementation) could provide a simple, inexpensive, and safe prevention strategy for type 2 diabetes. Resolution of this uncertainty is therefore important.

In this study, our main aim was to estimate the unconfounded, causal association between 25(OH)D concentrations and risk of type 2 diabetes using a mendelian randomisation approach, combining data from several studies. We also examined causal associations between 25(OH)D concentrations and glycaemic traits as secondary outcomes.

## Methods

### Study design

Based on the genome-wide associations previously identified,^8^ we used four SNPs as primary genetic instruments: rs12785878 near *DHCR7*, rs10741657 near *CYP2R1*, rs4588 of *DBP*, and rs17217119 near *CYP24A1*. Mendelian randomisation analysis requires genetic variants to be related to a main exposure, but not to potential confounders.^13^, ^14^ Thus, we first assessed the association of each of the four SNPs with 25(OH)D concentrations and other baseline factors (eg, BMI, blood pressure, and physical activity). Second, we examined the association between each SNP and risk of type 2 diabetes. Third, we combined these findings to estimate the unconfounded, causal association between 25(OH)D concentrations and risk of type 2 diabetes by mendelian randomisation analysis. Fourth, we also did mendelian randomisation analysis to estimate the unconfounded, causal association between 25(OH)D concentration and four glycaemic traits: concentrations of fasting glucose, 2-h glucose (during a standard oral glucose tolerance test), fasting insulin, and HbA_1c_. Finally, for comparison with the mendelian randomisation-based estimates, we meta-analysed data from prospective observational studies that assessed the association between 25(OH)D concentrations and risk of type 2 diabetes.

### Data sources for mendelian randomisation analysis

**Figure 1:**
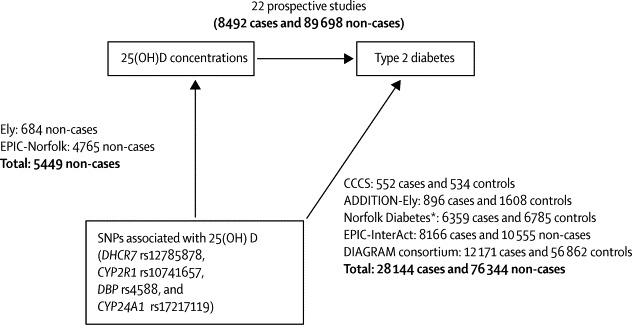
Study design and mendelian randomisation analysis of SNPs associated with 25(OH)D concentrations and risk of type 2 diabetes Because of some missing genotyping data for each single nucleotide polymorphism (SNP), the sample sizes for each study varies; total sample sizes are shown. CCCS=Cambridgeshire case-control study. *Excludes data from EPIC-Norfolk already included in EPIC-InterAct.

We used several data sources from populations of European descent (figure 1, appendix pp 2–5).^15^ We derived associations of SNPs with 25(OH)D concentrations using data from the Ely^16^ (n=684) and EPIC-Norfolk^4^ (n=4765) studies, assessing randomly selected adults with SNPs and 25(OH)D data available. 56 participants from the Ely study and 2020 from the EPIC-Norfolk study were excluded because data were not available. Associations of SNPs with risk of type 2 diabetes were based on 28 144 cases of type 2 diabetes and 76 344 non-cases from a case-cohort study (EPIC-InterAct)^17^ and four case-control studies (the DIAGRAM consortium,^18^ ADDITION-Ely,^19^, ^20^ Norfolk Diabetes,^15^ and Cambridgeshire^15^). Among participants assessed for SNPs, up to 4·5% were excluded because of missing genetic information. Some datasets included some overlap of study participants, but we ensured that these were not double-counted (appendix pp 2-4).^15^ Such overlap was present between the ADDITION-Ely and Ely studies, and between the Norfolk Diabetes, EPIC-Norfolk, and EPIC-InterAct studies. Apart from a few studies in DIAGRAM, all studies ascertained type 2 diabetes cases through biochemical testing (concentrations of fasting glucose, 2-h glucose, or HbA_1c_), diabetes registries, or medical records (appendix p 3). For associations between SNPs and glycaemic traits, we assessed summary data from 46 368 Europeans without diabetes in the Meta-Analyses of Glucose and Insulin-related traits Consortium (MAGIC)^21^. Ethical approval was obtained for all studies included in this analysis.

### Measurement of 25(OH)D concentration and SNPs

Circulating concentrations of 25(OH)D in the Ely^16^ and EPIC-Norfolk^4^ studies were measured as previously reported. The four SNPs included in our analysis (or SNPs in linkage disequilibrium, with *r*^2^>0·99) were previously identified as significant determinants of 25(OH)D concentration,^2^, ^8^, ^9^, ^10^, ^11^, ^22^ and data for these SNPs were also available for DIAGRAM and MAGIC.

In EPIC-InterAct, SNPs were assayed by Illumina 660 quad-chip (Illumina, Little Chesterford, UK; n=9343) at the Wellcome Trust Sanger Institute (Hinxton, UK), and Metabochip (Illumina; n=9381) at the Department of Pathology, University of Cambridge, Cambridge, UK. For the other studies (Ely, EPIC-Norfolk, Cambridgeshire, ADDITION-Ely, and Norfolk Diabetes), SNPs were genotyped with Custom TaqMan SNP Genotyping Assays (Applied Biosystems, Paisley, UK) at the Medical Research Council Epidemiology Unit, Cambridge, UK. The assays used 10 ng of genomic DNA in a 2·5 μl reaction volume, 384-well plate with a G-Storm GS4 Thermal Cycler (GRI, Rayne, UK). Endpoint detection and allele calling were done with an ABI PRISM 7900HT Sequence Detection System (Applied Biosystems, Paisley, UK). All assays passed the quality-control criteria (call rate greater than 95% and blind duplicate concordance of 97% or higher) and the frequency of SNPs was in accordance with Hardy–Weinberg equilibrium (p>0·05 among adults without diabetes in each study).

### Meta-analysis of 25(OH)D concentration and type 2 diabetes

We did a random-effects meta-analysis of the association between 25(OH)D concentrations and type 2 diabetes in prospective observational studies, updating a previous meta-analysis^4^ with a search for additional publications from Jan 31, 2012, to June 17, 2014 (appendix pp 4, 12–14). We only included studies in which participants were of European descent for comparability with our mendelian randomisation analysis.

### Statistical analyses

We assumed each SNP to have an additive effect on 25(OH)D concentration and risk of type 2 diabetes, as previously verified.^8^, ^10^ We first assessed the association of each SNP with 25(OH)D concentration using linear regression in the control participants of the Ely and EPIC-Norfolk studies, assuming a linear effect of each SNP per additional allele on 25(OH)D concentration and an additive model across SNPs, with adjustment for age, sex, and season of blood draw. We assessed the strength of associations using Cragg-Donald F-statistics, with values greater than 10 regarded as useful for mendelian randomisation analysis.^14^ Among adults without diabetes in prospective and case-control studies, we examined whether each SNP as an instrumental variable fulfilled the assumption of mendelian randomisation analysis that a SNP has no association with potential confounders.^14^, ^23^

As components of mendelian randomisation analysis (figure 1), we examined associations of each SNP with risk of type 2 diabetes, assuming a linear effect of each SNP on the logit of disease risk (in logistic models) or on the linear predictor of disease risk (in the Cox model) per additional variant allele, and an additive model across SNPs, with adjustment for age, sex, and BMI. We used logistic regression for the case-control studies and Prentice-weighted Cox regression for EPIC-InterAct (case-cohort study).^24^ We chose to adjust for BMI because DIAGRAM included study-specific estimates after adjustment for BMI^18^ and because body size might account for non-specific genetic and biological variation of 25(OH)D—eg, by diluting blood 25(OH)D concentrations.^25^

For the main mendelian randomisation analysis, we used a Bayesian likelihood-based method to estimate the unconfounded association of genetically predicted concentrations of 25(OH)D with risk of type 2 diabetes.^14^ This method quantitatively combined summary estimates for SNPs, including DIAGRAM data, rather than requiring individual-level data from each study.^14^ For each SNP, we fitted a model using WinBUGS,^26^ incorporating both measures of the association (β coefficients and SEs) between SNP and 25(OH)D concentration and measures of the association between SNP and risk of type 2 diabetes.

The model assumed a linear relationship between 25(OH)D concentrations and log odds of type 2 diabetes, and a bivariate normal distribution for the estimates of SNP–25(OH)D association and of SNP–type 2 diabetes association from each study. A bivariate model is assumed to allow for correlation between the genetic estimates with 25(OH)D concentration and with risk of type 2 diabetes when they are estimated in a single study for the same participants. Calculation of the unconfounded estimate for a 1 SD lower 25(OH)D concentration relies on a linearity assumption that extrapolates beyond the genetic data; however, this issue affects only the size of the estimate and not its significance.^23^ An estimate of the log odds ratio (OR) was scaled to be per 1 SD of 25(OH)D concentration (25·0 nmol/L), a weighted average of the SDs in observational studies included in meta-analysis. Additionally, we did separate mendelian randomisation analyses using allelic scores for SNPs for 25(OH)D synthesis (*DHCR7* and *CYP2R1*) and metabolism (*DBP* and *CYP24A1*).^2^, ^8^, ^9^, ^10^, ^11^, ^12^ We also did a sensitivity analysis in which we combined two previously reported mendelian randomisation-based estimates^9^, ^11^ (not including the EPIC-Potsdam study,^10^ which is a part of EPIC-InterAct).

We used a similar approach to estimate the unconfounded association of 25(OH)D concentrations with concentrations of fasting glucose, 2-h glucose, fasting insulin, and HbA_1c_. Measures of the SNP–25(OH)D association were the same as those used in the mendelian randomisation analysis for risk of type 2 diabetes. Measures of the SNP–trait associations were taken from MAGIC (appendix p 6), with linear regression used for the association analysis; concentration of fasting insulin was log-transformed.^21^

Analyses were done with Stata/SE13.1, unless otherwise stated. All statistical tests were two-sided (α=0·05).

### Role of the funding source

The funders had no role in study design, data collection, data analysis, data interpretation, or writing of the report. The corresponding author had full access to all the data in the study and had final responsibility for the decision to submit for publication.

## Results

In the studies that contributed data to the mendelian randomisation analysis to investigate causal associations of 25(OH)D concentration with type 2 diabetes and glycaemic traits (appendix p 5), most adults were aged 50–60 years and proportions of women ranged from 35% to 65%. Minor allele frequencies of SNPs were consistent across the studies: 0·39–0·41 for *CYP2R1* rs10741657, 0·24–0·29 for *DHCR7* rs12785878, 0·28–0·30 for *DBP* rs4588, and 0·21–0·22 for *CYP24A1* rs17217119.

**Figure 2:**
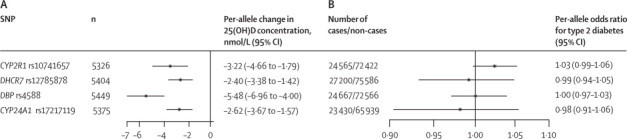
Associations of SNPs related to vitamin D metabolism with circulating concentrations of 25(OH)D (A) and risk of type 2 diabetes (B) For analysis of 25-hydroxyvitamin D (25[OH]D), summary estimates were obtained from adults without diabetes from two studies, Ely^16^ and EPIC-Norfolk.^4^ For analysis of type 2 diabetes, summary estimates were obtained from four case-control studies (Cambridgeshire,^15^ ADDITION-Ely,^19^, ^20^ Norfolk Diabetes,^15^ and DIAGRAM^18^) and one case-cohort study (EPIC-InterAct^17^). SNPs=single nucleotide polymorphisms.

All four SNPs were associated with 25(OH)D concentrations (figure 2A; appendix pp 7–9). Concentrations of 25(OH)D per risk allele (lowering 25[OH]D concentrations) were lower by 3·22 nmol/L (95% CI 1·79–4·66) for *CYP2R1* rs10741657, 2·40 nmol/L (1·42–3·38) for *DHCR7* rs12785878, 5·48 nmol/L (4·00–6·96) for *DBP* rs4588, and 2·62 nmol/L (1·57–3·67) for *CYP24A1* rs17217119. There was no clear evidence for pleiotropic effects of SNPs since they were not associated with other baseline factors such as BMI, blood pressure, or physical activity (appendix pp 7–8). The Cragg-Donald F-statistic ranged from 19·2 to 113·3 across individual SNPs and was 191·4 for a score in which the four SNPs were combined (appendix p 10). Each SNP accounted for 0·4–2·0% of the variation in 25(OH)D concentrations, and the four SNPs together accounted for 3·6% of the variation (appendix p 10).

The SNPs were not significantly associated with risk of type 2 diabetes (figure 2B, appendix p 10). Per 25(OH)D-lowering allele, ORs were 1·03 (95% CI 0·99–1·06) for *CYP2R1* rs10741657, 0·99 (0·94–1·05) for *DHCR7* rs12785878, 1·00 (0·97–1·03) for *DBP* rs4588, and 0·98 (0·91–1·06) for *CYP24A1* rs17217119.

**Figure 3:**
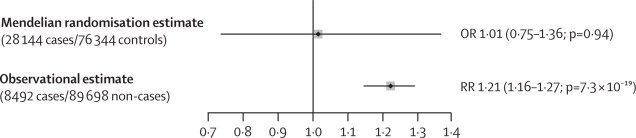
Mendelian randomisation and observational estimates for effect of a 1 SD reduction in 25(OH)D concentration on risk of type 2 diabetes The mendelian randomisation estimate (odds ratio [OR] with 95% CIs) was determined by instrumental variable analysis and the observational estimate (relative risk [RR] with 95% CIs) was determined by meta-analysis of prospective observational studies. 1 SD of 25-hydroxyvitamin D (25[OH]D) concentration was 25·0 nmol/L.

Mendelian randomisation analysis showed no significant associations of 25(OH)D with risk of type 2 diabetes risk. The summary OR per 1 SD lower 25(OH)D concentration was 1·01 (95% CI 0·75–1·36; p=0·94; figure 3). Similarly, the results were not significant for individual alleles or any allelic scores (appendix p 11). In the sensitivity analysis in which we combined two previously reported mendelian randomisation-based estimates,^9^, ^11^ the results were unchanged: OR per 1 SD lower 25(OH)D concentration was 1·04 (95% CI 0·82–1·31) for the four SNPs combined, 1·16 (0·95–1·41) for the two SNPs related to 25(OH)D synthesis, and 0·95 (0·64–1·42) for the two SNPs related to 25(OH)D metabolism.

In the secondary mendelian randomisation analysis for glycaemic traits, we identified no significant causal associations for the four SNPs combined or for two SNPs related to 25(OH)D metabolism (table). For the two SNPs related to 25(OH)D synthesis, we noted a significant causal association for one of the four traits (table).

**Table:** Mendelian randomisation estimates of the association between 1 SD lower 25(OH)D concentrations and glycaemic traits

	**n**	**Effect size (95% CI)**	**p value**
**All four SNPs (*CYP2R1* rs10741657, *DHCR7* rs12785878, *DBP* rs4588, and *CYP24A1* rs17217119)**			
Fasting glucose (mmol/L)	46 186	−0·02 (−0·04 to 0·01)	0·28
2-h glucose (mmol/L)	15 234	0·08 (−0·06 to 0·22)	0·25
Fasting insulin (% difference)*	46 186	−1·04 (−3·91 to 1·83)	0·48
HbA_1c_ (%)	46 368	0·01 (−0·04 to 0·05)	0·80
**Two SNPs near genes related to 25(OH)D synthesis (*CYP2R1* rs10741657 and *DHCR7* rs12785878)**			
Fasting glucose (mmol/L)	46 186	−0·03 (−0·08 to 0·02)	0·21
2-h glucose (mmol/L)	15 234	0·11 (−0·13 to 0·35	0·36
Fasting insulin (% difference)*	46 186	−5·44 (−10·64 to −0·24)	0·04
HbA_1c_ (%)	46 368	0·02 (−0·03 to 0·06)	0·48
**Two SNPs within or near genes related to 25(OH)D metabolism (*DBP* rs4588 and *CYP24A* 1 rs17217119)**			
Fasting glucose (mmol/L)	46 186	−0·01 (−0·04 to 0·03)	0·70
2-h glucose (mmol/L)	15 234	0·07 (−0·11 to 0·24)	0·44
Fasting insulin (% difference)*	46 186	1·46 (−2·13 to 5·06)	0·43
HbA_1c_ (%)	46 368	−0·03 (−0·06 to 0·01)	0·11

For single nucleotide polymorphisms (SNPs) and glycaemic traits, summary data from MAGIC^21^ were used; for SNPs and 25-hydroxyvitamin D (25[OH]D) concentrations, data from the Ely^16^ and EPIC-Norfolk^4^ studies were used. Sample sizes varied between SNPs. 1 SD of 25[OH]D concentration was 25·0 nmol/L.

* Effect size is presented as a percentage difference in log-transformed fasting insulin concentration.

**Figure 4:**
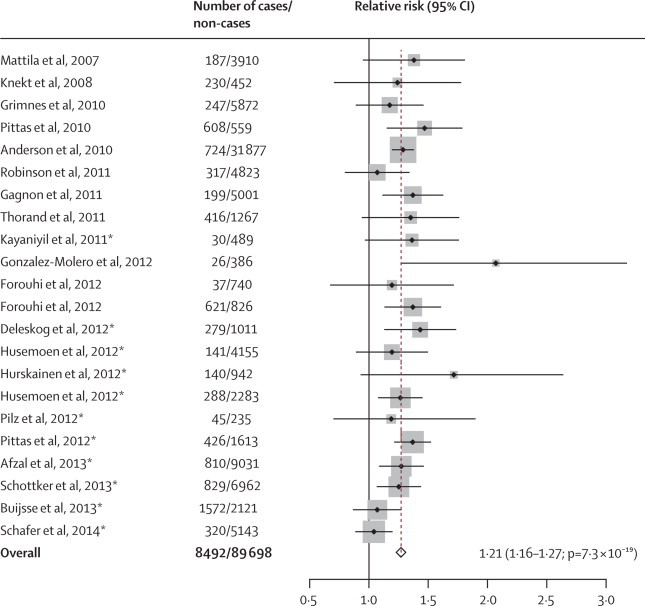
Meta-analysis of 22 prospective studies for associations of 1 SD lower 25(OH)D concentration with risk of type 2 diabetes in populations of European descent 1 SD of 25-hydroxyvitamin D (25[OH]D) concentration was 25·0 nmol/L. *Indicates studies added in this updated analysis (references 17–27 in appendix); other studies were included in our previous meta-analysis.^4^

In observational studies, investigators have previously identified an inverse association of 25(OH)D concentrations with risk of type 2 diabetes. We meta-analysed data from the 11 studies included in our previous meta-analysis^4^ and 11 newly identified studies (appendix pp 12–14). Based on this updated meta-analysis of 22 studies (8492 cases of type 2 diabetes and 89 698 non-cases), a 1 SD (25·0 nmol/L) lower 25(OH)D concentration was associated with an increased risk of type 2 diabetes (relative risk 1·21, 95% CI 1·16–1·27; p=7·3 × 10^−19^; Figure 3, Figure 4). We noted little evidence of heterogeneity (*I*^2^=18%, 95% CI 0–51; p=0·22) or publication bias (Begg's test p=0·48).

## Discussion

Although observational evidence from prospective studies suggests an association between low concentrations of 25(OH)D and an increased risk of incident type 2 diabetes, the results of our mendelian randomisation analysis suggest that genetically predicted low concentrations of 25(OH)D were not associated with risk of type 2 diabetes. This finding suggests that the association between 25(OH)D concentration and type 2 diabetes might not be causal (panel).

Panel **Research in context**

**Systematic review**

We searched PubMed for reports of relevant studies published in any language before June 17, 2014, about 25-hydroxyvitamin D (25[OH]D), a marker of vitamin D status, and incident type 2 diabetes, using search terms related to vitamin D concentrations (“25-hydroxy vitamin D” or “25(OH)D” or “vitamin D”) and diabetes outcomes (“diabetes” or “glucose” or “metabolic syndrome” or “hyperglycaemia”). We also searched PubMed for reports of mendelian randomisation studies, published in any language up to Aug 4, 2014, for the association of 25(OH)D concentrations and type 2 diabetes, using search terms related to mendelian randomisation studies (“mendelian randomisation” or “mendelian randomization”), vitamin D concentrations (“25-hydroxy vitamin D” or “25(OH)D” or “vitamin D”), and type 2 diabetes outcomes (“type 2 diabetes” or “T2D”). We reviewed reference lists of articles identified during the searches. We identified three studies^9^, ^10^, ^11^ that examined causal associations between 25(OH)D concentrations and risk of type 2 diabetes. Although results of observational studies suggested that low circulating concentrations of 25(OH)D were associated with an increased risk of type 2 diabetes, our search showed that previous evidence for the causal association between 25(OH)D and type 2 diabetes was limited by insufficient power, too few single-nucleotide polymorphisms, or absence of validation for intermediate risk factors for type 2 diabetes including glycaemic traits.

**Interpretation**

We identified a discordance between the observed inverse association and the null association estimated from mendelian randomisation analysis with four relevant genetic variants as instruments for the association between circulating 25(OH)D concentrations and risk of type 2 diabetes. This finding suggests that there is no causal link between 25(OH)D concentration and risk of type 2 diabetes, and that the observed inverse association might be subject to residual confounding and reverse causation bias. Our mendelian randomisation study was large and generated more precise mendelian randomisation estimates than did previous research.^9^, ^10^, ^11^ We also identified no causal associations between 25(OH)D concentrations and any glycaemic traits investigated, confirming our main finding. Together with existing reports of no benefit of vitamin D supplementation for reducing the incidence of type 2 diabetes,^6^, ^7^ our null results suggest that efforts to increase concentrations of 25(OH)D might not reduce the risk of type 2 diabetes, as would be expected from observational evidence.

Our finding by mendelian randomisation analysis was consistent with results from two clinical trials, the Women's Health Initiative^6^ and RECORD.^7^ Findings from neither trial showed a benefit for risk of type 2 diabetes with vitamin D supplementation in doses of 800 IU per day (20 μg per day) or less,^6^, ^7^ although these doses are probably too low for the potential effect of vitamin D to be seen.^3^ Our findings for glycaemic traits were also consistent with findings from clinical trials of vitamin D supplements and glycaemic outcomes, including glucose tolerance and HbA_1c_ concentration.^1^, ^3^

Mendelian randomisation analyses for 25(OH)D and risk of type 2 diabetes have been done for populations from Germany^10^ and Denmark.^11^ Investigators of neither study produced robust evidence of a causal association between 25(OH)D concentration and risk of type 2 diabetes. The investigators of the German study^10^ reported an OR of 1·10 (95% CI 0·68–1·79; p=0·70) per 25·0 nmol/L lower concentration of 25(OH)D (rescaled from the original presentation of a 5 nmol/L reduction), based on a genetic score derived from SNPs within or near five genes related to vitamin D metabolism. Investigators of the Danish study^11^ reported no significant association for each of two SNPs near *CYP2R1* and two SNPs near *DHCR7*, although use of a combined score of two SNPs near *DHCR7* provided weak causal evidence (p trend=0·04): the hazard ratio of the highest to the lowest genetic scores for lowering 25(OH)D concentrations (ie, the greatest to least 25[OH]D-lowering effect) was 1·11 (95% CI 0·96–1·29; p=0·17).

In a Norwegian study,^9^ the investigators reported significant associations of 12 SNPs related to vitamin D metabolism with 25(OH)D concentration, but not with type 2 diabetes.^9^ Consistent with the null findings for any of the individual SNPs, our findings also do not support a causal association between 25(OH)D concentration and risk of type 2 diabetes, with even greater precision than the Norwegian study. Moreover, the findings from our mendelian randomisation analysis suggest that 25(OH)D concentrations are associated with neither increased nor decreased risk of type 2 diabetes. This finding is supported by the results of our mendelian randomisation analysis of 25(OH)D concentrations and glycaemic traits (to our knowledge, the first such analysis that has been reported), which showed no causal association.

Our findings challenge some views about the biological roles of vitamin D. Although the direct mechanisms are unclear, previous findings have suggested roles for vitamin D in type 2 diabetes.^1^, ^3^ For example, results of rodent experiments showed that 1,25-dihydroxyvitamin D (1,25[OH]_2_D), an active form of vitamin D, regulates calcium-dependent signalling, modulates insulin secretion in pancreatic β cells, and reduces lipotoxicity of free fatty acids.^3^ Notably, concentrations of 25(OH)D and 1,25(OH)_2_D are weakly correlated (*r*<0·3).^27^ Thus, our mendelian randomisation analysis and others^10^, ^11^ might be limited in their ability to elucidate a causal role of biologically active vitamin D. This finding should therefore stimulate future research into 1,25(OH)_2_D, which remains understudied because of its short half-life (12–36 h *vs* 3 weeks for 25[OH]D) and its low concentration in blood (less than 1% of the concentration of 25[OH]D).^27^

Cautious interpretation is also necessary because of the roles of genes related to vitamin D metabolism. The *DBP* gene encoding vitamin D-binding protein, involved in 25(OH)D transport, is an unconfounded determinant of 25(OH)D concentration, but might have an opposing genetic effect on 1,25(OH)_2_D. The *DBP* gene product associated with increased 25(OH)D concentrations might sequester 25(OH)D in blood and reduce bioavailability of 25(OH)D. This possibility has been supported by evidence from Powe and colleagues,^22^ who reported that *DBP* alleles that increased blood concentrations of 25(OH)D were associated with reduced bone mineral density. Therefore, 25(OH)D represents vitamin D status, but not necessarily the amount of bioavailable vitamin D. Thus, existing mendelian randomisation analyses, including ours, cannot to accurately predict amounts of bioavailable vitamin D. This possibility and the potential of mendelian randomisation analysis to be used to investigate bioavailable 25(OH)D, as well as circulating vitamin D-binding protein and 1,25(OH)_2_D, need to be studied. Moreover, functions independent of diabetes aetiology should also be investigated in the future, since vitamin D-binding protein might have diverse anti-inflammatory functions and results of mendelian randomisation analyses have supported a causal antihypertensive role of vitamin D.^2^, ^28^

Our analysis has several strengths. It is the largest mendelian randomisation analysis for 25(OH)D concentrations and risk of type 2 diabetes, with four times as many cases of type 2 diabetes as previous research.^9^, ^10^, ^11^ Furthermore, it includes a novel mendelian randomisation analysis of four glycaemic traits and an updated meta-analysis of observational studies assessing 25(OH)D concentration and risk of type 2 diabetes.

Our study also has limitations. Our mendelian randomisation analyses and meta-analysis of observational studies combined data from several studies to maximise power, which could introduce bias due to different study designs that can be difficult to quantify with respect to the definition of endpoints and measurement of exposures. Despite no evidence of such bias, we cannot rule it out as an explanation for the null finding. The four SNPs investigated in this study account for only 3·6% of the variation in 25(OH)D concentration, as previously reported.^8^, ^10^ This limitation could be minimised by examining several SNPs from a single gene or from the whole genome as polygenic effects, although increasing the diversity of the SNP panel also brings increased potential for pleiotropic effects.^2^, ^11^, ^29^ Another limitation is that the use of total 25(OH)D concentration cannot distinguish between endogenous 25(OH)D_3_ and exogenous vitamin D, including 25(OH)D_2_. Future research to separate the two should improve genetic evidence further. Additionally, we assumed linear associations between SNPs, 25(OH)D concentrations, and risk of type 2 diabetes, and did not examine different physiological ranges of 25(OH)D concentration. Threshold effects of genes on 25(OH)D concentration and of 25(OH)D concentration on risk of type 2 diabetes could be present, for example, depending on amounts of sunlight exposure and latitudes of residence. Future detailed analyses are warranted, including stratification by demographic characteristics and a range of 25(OH)D concentrations. Finally, because we studied only white European populations, our findings might not be generalisable to other ethnic groups.

Our findings suggest that interventions to reduce the risk of type 2 diabetes by increasing concentrations of 25(OH)D are not currently justified. Rather, our findings emphasise the need for investigation of the discrepancy between the observational evidence and the absence of causal evidence. Such discrepancy could be accounted for by possible residual confounding in observational studies that cannot fully control for confounding. For example, physical activity might be a strong confounder, because it reduces risk of type 2 diabetes but is independently related to sunlight exposure and therefore vitamin D status. Existing observational studies generally recorded self-reported physical activity, which is subject to errors and residual confounding. To resolve this issue, research in which physical activity is measured objectively—eg, with accelerometers—will be needed to minimise potential confounding.^5^

Adiposity might also confound the association substantially. Increased adiposity both lowers 25(OH)D concentration and increases the risk of type 2 diabetes, as the results of previous mendelian randomisation studies have shown.^11^, ^12^ Most observational studies adjusted for measures of adiposity, including BMI, but this adjustment might also involve measurement error, allowing residual confounding. Additionally, the discrepancy between findings of observational and mendelian randomisation studies suggest reverse causality. For example, subclinical diseases such as liver disease can lower production of 25(OH)D as well as increase the risk of type 2 diabetes.^30^ Overall, underlying bias might account for the discrepancy between observational and causal evidence, justifying further research into detailed measurements of vitamin D exposure, more precise estimates of lifestyle factors, and subclinical characteristics. Such research will help us to identify causal factors that increase 25(OH)D concentration and also reduce the risk of type 2 diabetes.

**This online publication has been corrected. The corrected version first appeared at thelancet.com/diabetes-endocrinology on December 11, 2014**
