# Supplementary material for: Association between circulating 25-hydroxyvitamin D and incident type 2 diabetes: a mendelian randomisation study
Source: Lancet Diabetes Endocrinol. 2015 Jan;3(1):35–42. doi: 10.1016/S2213-8587(14)70184-6 (PMC4286815; doi:10.1016/S2213-8587(14)70184-6)
Supplement: Supplementary appendix [file mmc1.pdf]

THE LANCET Diabetes & Endocrinology

Supplementary appendix

This appendix formed part of the original submission and has been peer reviewed. We post it as supplied by the authors.

Supplement to: Ye Z, Sharp SJ, Burgess S, et al. Association between circulating 25-hydroxyvitamin D and incident type 2 diabetes: a mendelian randomisation study. *Lancet Diabetes Endocrinol* 2014; published online Oct 1. http://dx.doi.org/10.1016/S2213-8587(14)70184-6.

This supplementary appendix has been corrected. The corrected version first appeared at thelancet.com/diabetes-endocrinology on Dec 11, 2014

Online Supplementary Materials

Supplementary Text ... 2
Descriptions of studies contributing to mendelian randomisation analysis ... 2
Searching strategy and meta-analysis of prospective studies on circulating 25-hydroxy vitamin D and incident type 2 diabetes ... 4
Supplementary Table 1. Characteristics of participants in the studies for analysis on associations of single nucleotide polymorphisms with type 2 diabetes (T2D) risk and circulating 25-hydroxy vitamin D. ... 5
Supplementary Table 2. Association of four vitamin D-related single nucleotide polymorphisms with fasting glucose, 2-hour glucose, fasting insulin, and HbA1c: Meta-Analyses of Glucose and Insulin-related traits Consortium (MAGIC). ... 6
Supplementary Figure 1. Association of four single nucleotide polymorphisms with 25-hydroxy vitamin D concentrations, their genetic scores, and various characteristics assessed in non-diabetic adults in four studies: control participants from Cambridgeshire case-control study, non-diabetic adults from the Ely study, EPIC-Norfolk study, and the EPIC-InterAct study ... 7
Supplementary Figure 2. Meta-analysis of the associations between four vitamin D-related single nucleotide polymorphisms and 25-hydroxy vitamin D concentrations among non-diabetic adults in the Ely and EPIC-Norfolk studies. ... 9
Supplementary Table 3. F-statistics and variation in 25-hydroxy vitamin D concentrations related to four vitamin D-related single nucleotide polymorphisms ... 10
Supplementary Figure 3. Associations between four single nucleotide polymorphisms related to vitamin D metabolism and risk of type 2 diabetes ... 10
Supplementary Table 4. Mendelian randomisation estimates of the association between genetically predicted concentrations of 25-hydroxy vitamin D and risk of type 2 diabetes ... 11
Supplementary Figure 4. Flow diagram of literature search and identification of studies for the association between circulating concentrations of 25-hydroxy vitamin D and risk of type 2 diabetes ... 12
Supplementary Table 5. Prospective studies of the association of circulating concentrations of 25-hydroxy vitamin D with risk of type 2 diabetes, newly included in the updated meta-analysis. ... 13
References for online supplementary materials ... 15

Supplementary Text

Descriptions of studies contributing to mendelian randomisation analysis

Studies contributing to mendelian randomisation (MR) analysis are shown in Supplementary Table 1 and Supplementary Figure 1. In our MR analysis, there was some overlap of study participants who contributed to the multiple datasets, as highlighted by asterisk (*) in text and as conducted in previous MR analysis on type 2 diabetes (T2D).[1] We evaluated them in an appropriate fashion to avoid double-counting. Sample sizes for genetic analysis varied across single nucleotide polymorphisms (SNPs) and cohorts, due to a small number of missing genotyping data in each of our datasets (<4·5% for each SNP). We excluded individuals with missing information on genes and missing information on 25-hydroxy vitamin D biomarkers, 25(OH)D, or diagnosis of T2D, depending on the outcome in analysis. The numbers of individuals included in MR analysis are shown in Supplementary Figures 1, 3, and 4.

Studies contributing to estimates of associations between SNPs and concentrations of 25(OH)D.

The Ely study*: The Ely study is a population-based cohort of white European men and women in Ely, Cambridge, UK.[2–5] European-origin adults, aged 40–69 years and free of known diabetes and registered with a single practice serving Ely, were randomly selected. The baseline examination took place between 1990 and 1992. In the ADDITION-Ely case-control study (described below), controls were selected from the non-diabetic adults in Ely[1], where oral-glucose tolerance test was implemented at baseline and during the follow-up. Among 1,608 non-diabetic adults with genotype information, 25(OH)D measurements were undertaken among 684 adults.[5] All participants gave written informed consent.

EPIC-Norfolk study*: The EPIC (European Prospective Investigation of Cancer)-Norfolk study is a prospective cohort of 25,639 men and women, aged 40-79 years, who were recruited between 1993 and 1997 in Norfolk, UK.[5,6] A detailed description of the study design and cohort characteristics has previously been published.[5] In the current analyses, 6785 participants free of known diabetes at baseline or during follow-up were randomly selected for genotyping. Among them, 4765 participants had 25(OH)D data.[1] For 25(OH)D concentrations , ultra-performance liquid chromatography–tandem mass spectrometry was used to measure 25(OH) vitamin D_2 (present in very small concentration) and 25(OH) vitamin D_3, which were summed to provide a measure of total 25(OH) vitamin D.[5] Ethical approval for the study was obtained from the Norfolk and Norwich Hospital Ethics Committee.

Studies contributing to estimates of associations between SNPs and risk of T2D

Cambridgeshire case–control study (CCCS): the Cambridgeshire case–control study is a population based study of type 2 diabetes (T2D).[1,7] Diabetes cases aged 45-76 years were randomly selected from general practitioner (GP) diabetes registers in Cambridgeshire, UK, and T2D was defined as onset of diabetes after the age of 30 years and without insulin use in the first year after diagnosis. Controls were recruited at random from the same population sampling frame and individually matched to cases for age (years), sex and site of GP registry.[1] Controls were screened as non-diabetic by medical records and glycated haemoglobin not more than 6%. We excluded participants with missing information on body mass index (BMI) (n=15). In the current analyses, we included 552 T2D cases and 534 controls that had DNA available and information on BMI. The study received ethical approval from the Cambridge Local Research Ethics Committee, and participants provided informed consent.

ADDITION-Ely case-control study: Cases of this study was derived from the ADDITION-Cambridge study, a multi-centre intervention study which focuses on effectiveness of stepwise screening on morbidity and mortality among people with new onset T2D.[1,8] People aged 40-69 years at high-risk of undiagnosed diabetes in East Angelia region participated in the study. Written informed consent was obtained for all participants at the time of the diabetes screening appointment and subsequent diagnostic test. Among adults aged 40 to 69 year participating in the UK Cambridge arm of the ADDITION study, new onset T2D cases were identified via a population-based stepwise screening strategy, including casual glucose, plasma glucose, glycated haemoglobin, and oral-glucose tolerance test.[8] All T2D cases were confirmed by 75g oral-glucose tolerance test. The controls were identified from Ely Study through matching by age, sex, and GP registry (as seen above*****).[1] We excluded participants with missing data on BMI (n=11), or on age and/or sex (n=3), or non-white participants (n=30). Current analyses included 896 T2D cases and 1,608 controls of white European-origin men and women who had DNA available and information on BMI. The Cambridge Research Ethics Committee approved both studies.

Norfolk Diabetes case-control study: The Norfolk Diabetes case-control study is an ongoing study of white European men and women with T2D in Norfolk.[5,9] All diabetes patients identified through GP diabetes registers in Norfolk, local hospital diabetes clinic and retinal screening programme patient registers were invited to participate. European cases aged 30 years or older were included as cases in this study. T2D was defined by not treated with insulin during the first year of diagnosis. Those with cystic fibrosis, chronic pancreatitis or long term steroid use were excluded from the study. After excluding participants with missing data on sex (n=18), or non-white participants (n=277), a total of 6359 cases were included in the current analyses. Control participants (n = 6785) free of known diabetes at baseline or during follow-up were randomly selected from among EPIC-Norfolk participants (as seen above*****). Because 830 participants from the EPIC-Norfolk study were also part of the EPIC-InterAct study (see below**)**, we excluded those participants from the EPIC-Norfolk study to avoid double counting. The Norfolk study was approved by the Norwich Local Research Ethics Committee.

EPIC-InterAct study: The EPIC-InterAct study is a large prospective case-cohort study nested within the Europe-wide EPIC study, involving 27,779 individuals from eight European countries.[10] The EPIC-InterAct study comprises 12,403 incident cases of T2D derived from a total cohort of 340,234 individuals comprising 3·99 million person-years of follow-up and includes a representative sub-cohort (n=16,154) that also includes 778 of the 12,403 incident T2D cases (as per the design of a case-cohort study).[10] Ascertainment of incident T2D involved a review of the existing EPIC datasets at each centre using multiple sources of evidence. None of the cases were ascertained solely by self-reported diagnosis. Follow-up was censored at the date of diagnosis, the 31st of December 2007, or the date of death, whichever came first. After excluding participants with missing data on BMI (n=197), in the current analyses, we included 8166 incident cases of T2D and 10,555 diabetes free participants. All participants gave written informed consent, and the study was approved by the local ethics committees in the participating centres and the Internal Review Board of the International Agency for Research on Cancer.

DIAGRAM (DIAbetes Genetics Replication And Meta-analysis): The DIAGRAM consortium represents the effort of groups of international researchers working on the genetics of T2D, with a principal focus on sample of European descent. The details are available in a prior publication.[11] Summary data from DIAGRAM consortium (12,171 T2D cases and 56,862 controls) are available at www.diagram-consortium.org.

Studies contributing to estimates of associations between SNPs and glycaemic traits.

MAGIC (the Meta-Analyses of Glucose and Insulin-related traits Consortium): MAGIC represents a collaborative effort to combine data from multiple GWAS to identify additional loci that impact on glycaemic and metabolic traits. The details are available in prior publications.[12–14] Summary data from MAGIC (n=46,391) are available at www.magicinvestigators.org.

Searching strategy and meta-analysis of prospective studies on circulating 25-hydroxy vitamin D and incident type 2 diabetes

We updated our previous meta-analysis of the association between circulating 25-hydroxy vitamin D, 25(OH)D, and incident T2D.[5] We identified all the additional studies published between 31 January 2012 and 17 June 2014. Electronic searches were performed for publications in PubMed. We also reviewed reference lists of articles identified during the search. The search terms were related to vitamin D concentrations (“25-hydroxy vitamin D” or “25(OH)D” or “vitamin D”), and diabetes risk (“diabetes” or “glucose” or “metabolic syndrome” or “hyperglycaemia”) without limits on publication date or language.

We included prospective studies that measured circulating 25(OH)D and used standard definitions of T2D based on World Health Organization criteria.[5] Since MR analyses only included participants of European descent, the meta-analysis of prospective studies was also restricted to European individuals.

For each eligible study newly identified (not in our previous meta-analysis[5]), information was extracted based on a pre-specified protocol. We considered odds ratios, risk ratios, and hazard ratios as estimates of the relative risk. The prospective association of 25(OH)D concentrations with T2D was summarized using random effects meta-analysis. The overall effect was estimated for per 1 SD lower 25(OH)D concentration. Heterogeneity was assessed using the Q-statistic test[15] and the I^2 statistic[16]. Publication bias was assessed by Begg’s test.

Supplementary Table 1. Characteristics of participants in the studies for analysis on associations of single nucleotide polymorphisms with type 2 diabetes (T2D) risk and circulating 25-hydroxy vitamin D.*

Study*	Use in Mendelian randomisation analysis			Characteristics of participants			
	SNP and 25(OH)D concentrations	SNP and T2D risk	SNP and glycaemic & insulin traits	N	Women, %	Age, years	BMI, kg/m^2
**Ely study**							
Non-diabetic adults	X			684	59·9	63·3 (7·7)	26·9 (4·4)
**EPIC-Norfolk study**							
Non-diabetic adults	X			4765	51·7	59·1 (9·0)	26·0 (3·6)
**Cambridgeshire CC Study**							
T2D cases		X		552	36·1	63·4 (7·9)	29·8 (5·3)
Controls		X		534	35·4	63·8 (7·8)	27·4 (4·2)
**ADDITION-Ely CC Study**							
T2D cases		X		896	38·1	61·2 (7·2)	33·1 (5·7)
Controls		X		1608	55·1	60·9 (9·1)	27·1 (4·7)
**Norfolk Diabetes CC Study†**							
T2D cases		X		6359	41·1	68·0 (11·3)	30·0 (5·8)
Controls		X		6785	51·8	59·3 (9·3)	26·2 (3·8)
**EPIC-InterAct Study†**							
T2D cases		X		8166	52·1	55·3 (8·2)	29·9 (3·1)
Non-diabetic adults		X		10555	64·7	51·6 (7·3)	25·8 (4·1)
**DIAGRAM§**							
T2D cases		X		12171	47·6	60·4 (8·8)	29·1 (4·4)
Controls		X		56862	60·9	54·5 (13·7)	25·2 (4·3)
**MAGIC§**							
Non-diabetic adults			X	46391	56·0	51·8 (12·8)	26·4 (4·4)

Abbreviations: 25(OH)D, 25-hydroxy vitamin D; BMI, body-mass index; DIAGRAM, DIAbetes Genetics Replication And Meta-analysis; CC, case-control MAGIC: Meta-Analyses of Glucose and Insulin-related traits Consortium; SNP, single nucleotide polymorphism; T2D, type 2 diabetes.

* Ely study and EPIC-Norfolk study contributed to analysis on associations of SNPs with 25(OH)D. EPIC-Norfolk study only has 4765 participants with genotyping data. Four case-control studies and one case cohort study (EPIC-InterAct study) contributed to the analysis on associations of SNPs with type 2 diabetes risk. Due to some missing genotyping data for each SNPs, the sample sizes for each study may vary for the association of SNPs with 25(OH)D concentrations and T2D.

† The Norfolk Diabetes Case-Control Study and the EPIC-InterAct study partly comprised a subset of EPIC-Norfolk Study. Thus, EPIC-Norfolk Study did not contribute to the analysis of SNP-T2D risk. In the analysis of EPIC-InterAct, those from the EPIC-Norfolk study (Norfolk Diabetes Case-Control Study).

§ The characteristics were calculated, using the information available in published literature and online (http://diagram-consortium.org for DIAGRAM and http://www.magicinvestigators.org for MAGIC).[12–14] MAGIC provided information on associations of SNP with glycaemic traits among non-diabetic adults, not evaluating T2D cases.

Supplementary Table 2. Association of four vitamin D-related single nucleotide polymorphisms with fasting glucose, 2-hour glucose, fasting insulin, and HbA1c: Meta-Analyses of Glucose and Insulin-related traits Consortium (MAGIC).*

**Single nucleotide polymorphisms (Gene symbol)**	**Effect allele**	**Other allele**	**Effect**	**Standard error**	***P*-value**
Fasting glucose (mmol/L)					
rs10741657 (*CYP2R1*)	G	A	-0·0033	0·0037	0·37
rs12785878 (*DHCR7*)	G	T	-0·0039	0·0040	0·32
rs2282679† (*DBP*)	G	T	0·0000	0·0042	1·00
rs17217119†(*CYP24A1*)	G	A	-0·0041	0·0044	0·36
2-hr glucose (mmol/L) adjusted for body-mass index					
rs10741657 (*CYP2R1*)	G	A	0·032	0·019	0·093
rs12785878 (*DHCR7*)	G	T	-0·017	0·02	0·40
rs2282679† (*DBP*)	G	T	0·027	0·021	0·21
rs17217119†(*CYP24A1*)	G	A	-0·024	0·023	0·29
Fasting insulin, (pmol/L, log-transformed)					
rs10741657 (*CYP2R1*)	G	A	-0·0057	0·0038	0·14
rs12785878 (*DHCR7*)	G	T	-0·007	0·0041	0·091
rs2282679† (*DBP*)	G	T	0·0055	0·0044	0·22
rs17217119†(*CYP24A1*)	G	A	-0·0038	0·0046	0·41
HbA1c (%)					
rs10741657 (*CYP2R1*)	G	A	0·001	0·0035	0·77
rs12785878 (*DHCR7*)	G	T	0·0032	0·0038	0·40
rs2282679† (*DBP*)	G	T	-0·0067	0·0038	0·077
rs17217119†(*CYP24A1*)	G	A	0·0001	0·0041	0·98

* Available online (http://www.magicinvestigators.org).
†rs2282679 was used as a proxy for rs4588 (r^2>0·99); rs17217119, a proxy for rs6013897 (r^2>0·99).

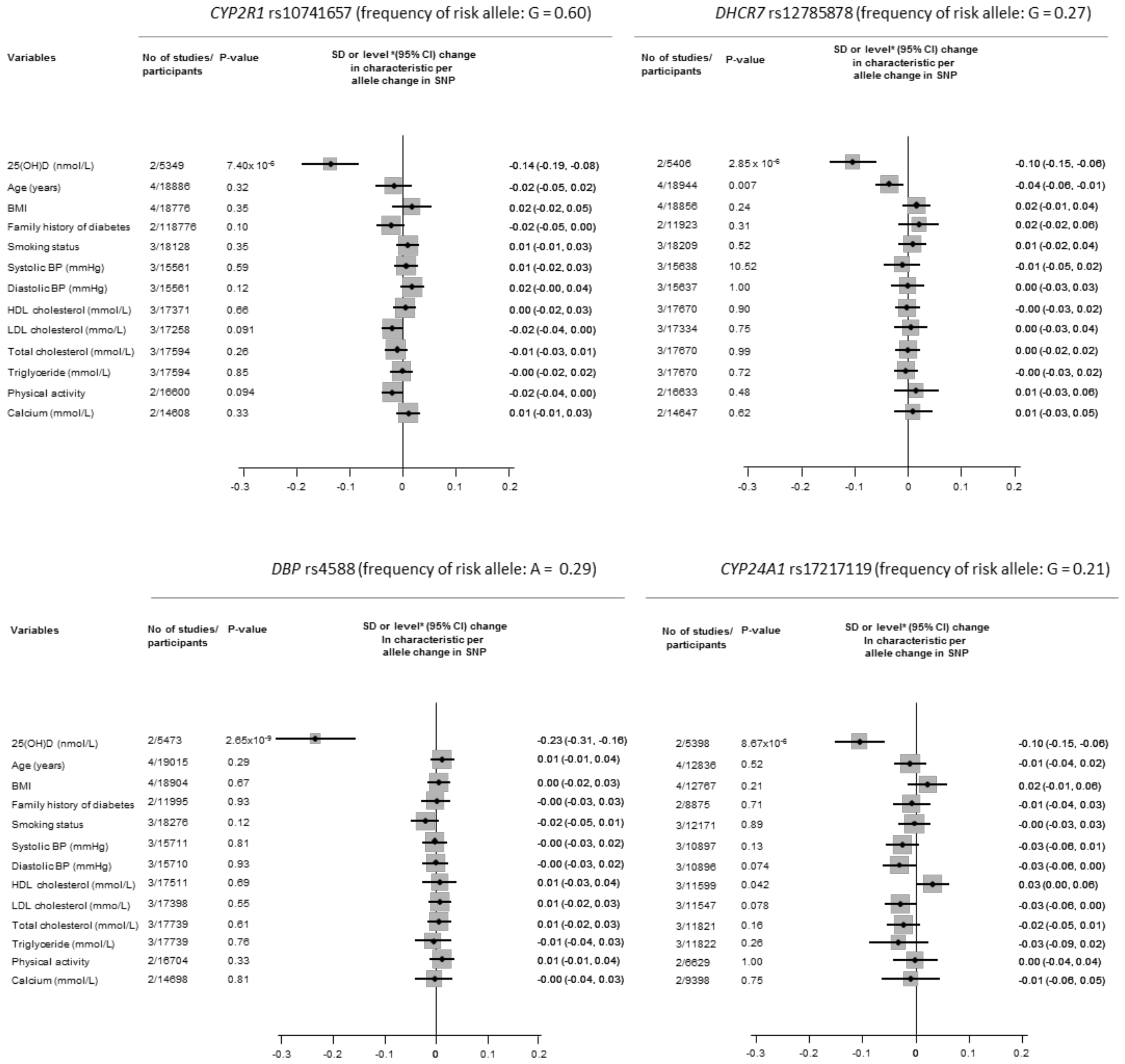

Supplementary Figure 1. Association of four single nucleotide polymorphisms with 25-hydroxy vitamin D concentrations, their genetic scores, and various characteristics assessed in non-diabetic adults in four studies: control participants from Cambridgeshire case-control study, non-diabetic adults from the Ely study, EPIC-Norfolk study, and the EPIC-InterAct study. Estimates were based on random-effects meta-analysis of study specific associations of each SNP with potential confounders. Four SNPs related to vitamin D synthesis and metabolism: *CYP2R1* rs10741657, *DHCR7* rs12785878, *DBP* rs4588, and *CYP24A1* rs17217119.
* SD changes for continuous variables and change in levels (based on polynomial logistic regression) for family history of diabetes (yes or no); smoking status (never smoked, ever smoker, or current smoker); or physical activity (inactive, moderately inactive, moderately active, and active).
Abbreviations: 25-hydroxy vitamin D, 25(OH)D; single nucleotide polymorphism, SNP; BMI, body-mass index; CI, confidence interval; HDL, high-density lipoprotein; LDL, low-density lipoprotein; SD, standard deviation.

Genetic score (*CYP2R1* rs10741657, and *DHCR7* rs12785878)

Variables	No of studies/ participants	P-value	SD or level* (95% CI) change in biomarker per allele change in gene score
25(OH)D	2/5261	$4.98x10^{-8}$	-0.06 (-0.08, -0.04)
Age (years)	4/18760	0.012	-0.01 (-0.02, -0.00)
BMI	4/18672	0.28	0.01 (-0.01, 0.02)
Family history of diabetes	2/11973	0.64	-0.00 (-0.01, 0.01)
Smoking status	3/18033	0.53	0.00 (-0.01, 0.01)
Systolic BP (mmHg)	3/15464	0.94	0.00 (-0.01, 0.01)
Diastolic BP (mmHg)	3/15463	0.23	0.00 (-0.00, 0.01)
HDL cholesterol (mmol/L)	3/17277	0.56	0.00 (-0.00, 0.01)
LDL cholesterol (mmol/L)	3/17164	0.29	-0.00 (-0.01, 0.00)
Total cholesterol (mmol/L)	3/17498	0.48	-0.00 (-0.01, 0.00)
Triglyceride (mmol/L)	3/17498	0.81	-0.00 (-0.01, 0.01)
Physical activity	3/16502	0.71	-0.00 (-0.02, 0.01)
Calcium (mmol/L)	2/14537	0.22	0.00 (-0.00, 0.01)

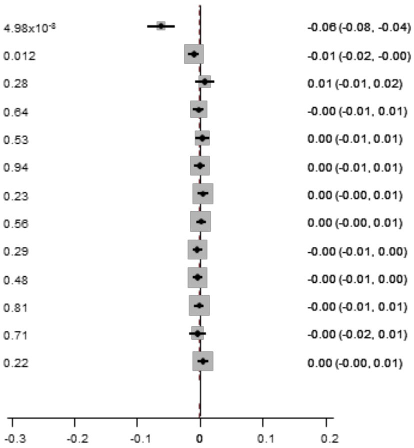

Genetic score (*DBP* rs4588, and *CYP24A1* rs17217119)

Variables	No of studies/ participants	P-value	SD or level* (95% CI) change in biomarker per allele change in gene score
25(OH)D	2/5326	$3.12x10^{-30}$	-0.08 (-0.10, -0.07)
Age (years)	4/12709	0.64	0.00 (-0.01, 0.01)
BMI	4/1640	0.66	0.00 (-0.01, 0.01)
Family history of diabetes	2/8781	0.53	-0.00 (-0.01, 0.01)
Smoking status	3/12077	0.26	-0.01 (-0.02, 0.01)
Systolic BP (mmHg)	3/10804	0.49	-0.00 (-0.01, 0.01)
Diastolic BP (mmHg)	3/10803	0.30	-0.00 (-0.01, 0.00)
HDL cholesterol (mmol/L)	3/11507	0.50	0.00 (-0.01, 0.02)
LDL cholesterol (mmol/L)	3/11455	0.55	-0.00 (-0.01, 0.01)
Total cholesterol (mmol/L)	3/11730	0.52	-0.00 (-0.01, 0.01)
Triglyceride (mmol/L)	3/11730	0.47	-0.01 (-0.02, 0.01)
Physical activity	3/11077	0.28	0.00 (-0.00, 0.01)
Calcium (mmol/L)	2/9322	0.78	-0.00 (-0.03, 0.02)

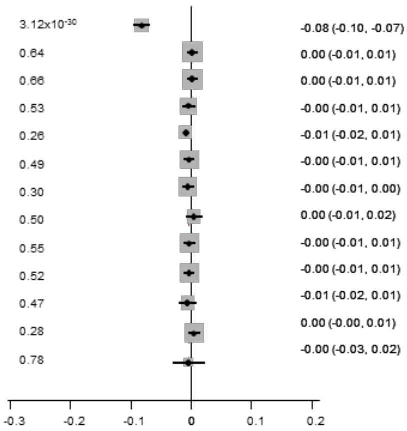

Genetic score (*CYP2R1* rs10741657, *DHCR7* rs12785878, *DBP* rs4588, and *CYP24A1* rs17217119)

Variables	No of studies/ participants	P-value	SD or level* (95% CI) change in biomarker per allele change in gene score
25(OH)D (nmol/L)	2/5130	$2.77x10^{-19}$	-0.14 (-0.17, -0.11)
Age (years)	4/18528	0.35	-0.00 (-0.01, 0.00)
BMI	4/18440	0.15	0.01 (-0.00, 0.02)
Family history of diabetes	2/11617	0.76	-0.00 (-0.01, 0.01)
Smoking status	3/17843	0.54	-0.00 (-0.02, 0.01)
Systolic BP (mmHg)	3/15278	0.62	-0.00 (-0.01, 0.01)
Diastolic BP (mmHg)	3/15277	0.78	0.00 (-0.01, 0.01)
HDL cholesterol (mmol/L)	3/17098	0.12	0.01 (-0.00, 0.02)
LDL cholesterol (mmo/L)	3/16985	0.53	-0.00 (-0.01, 0.01)
Total cholesterol (mmol/L)	3/17315	0.67	-0.00 (-0.01, 0.01)
Triglyceride (mmol/L)	3/17315	0.22	-0.01 (-0.02, 0.00)
Physical activity	2/16324	0.54	0.00 (-0.01, 0.02)
Calcium (mmol/L)	2/14396	0.81	0.00 (-0.01, 0.02)

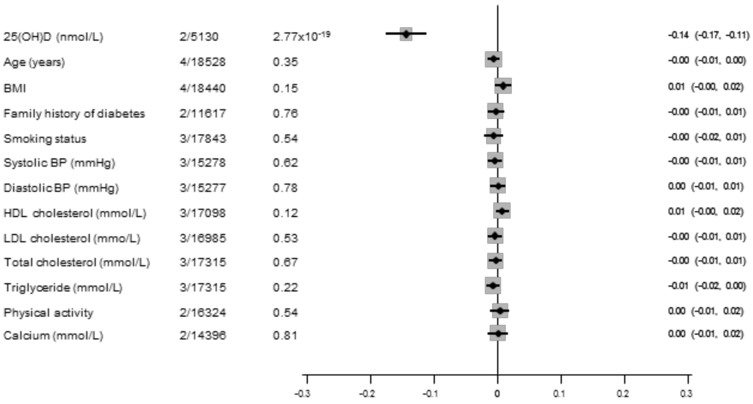

Supplementary Figure 1 (Continued).

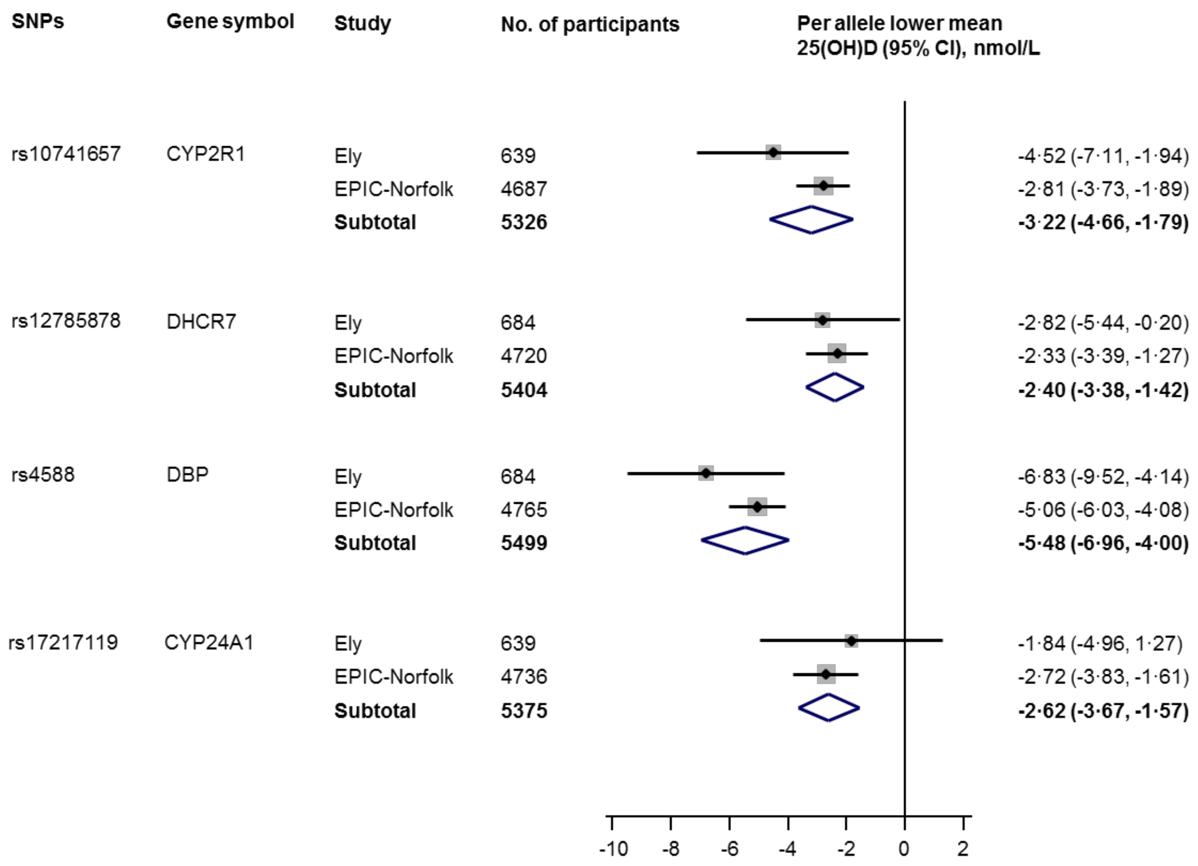

Supplementary Figure 2. Meta-analysis of the associations between four vitamin D-related single nucleotide polymorphisms and 25-hydroxy vitamin D concentrations among non-diabetic adults in the Ely and EPIC-Norfolk studies. Abbreviations: 25(OH)D, 25-hydroxy vitamin D; CI, confidence interval.

Supplementary Table 3. F-statistics and variation in 25-hydroxy vitamin D concentrations related to four vitamin D-related single nucleotide polymorphisms.*

Related function and gene symbol	SNP	F-statistics	Variation of 25(OH)D levels predicted by each SNP
Synthesis of 25(OH)D			
CYP2R1	rs10741657	44·2	0·8%
DHCR7	rs12785878	22·0	0·4%
Metabolism of 25(OH)D			
DBP	rs4588	113·3	2·0%
CYP24A1	rs17217119	19·2	0·4%
Four SNPs combined		191·4	3·6%

*The analysis only considered non-diabetic participants in the Ely and EPIC-Norfolk studies. Abbreviations: 25(OH)D, 25-hydroxy vitamin D; SNP, single nucleotide polymorphism.

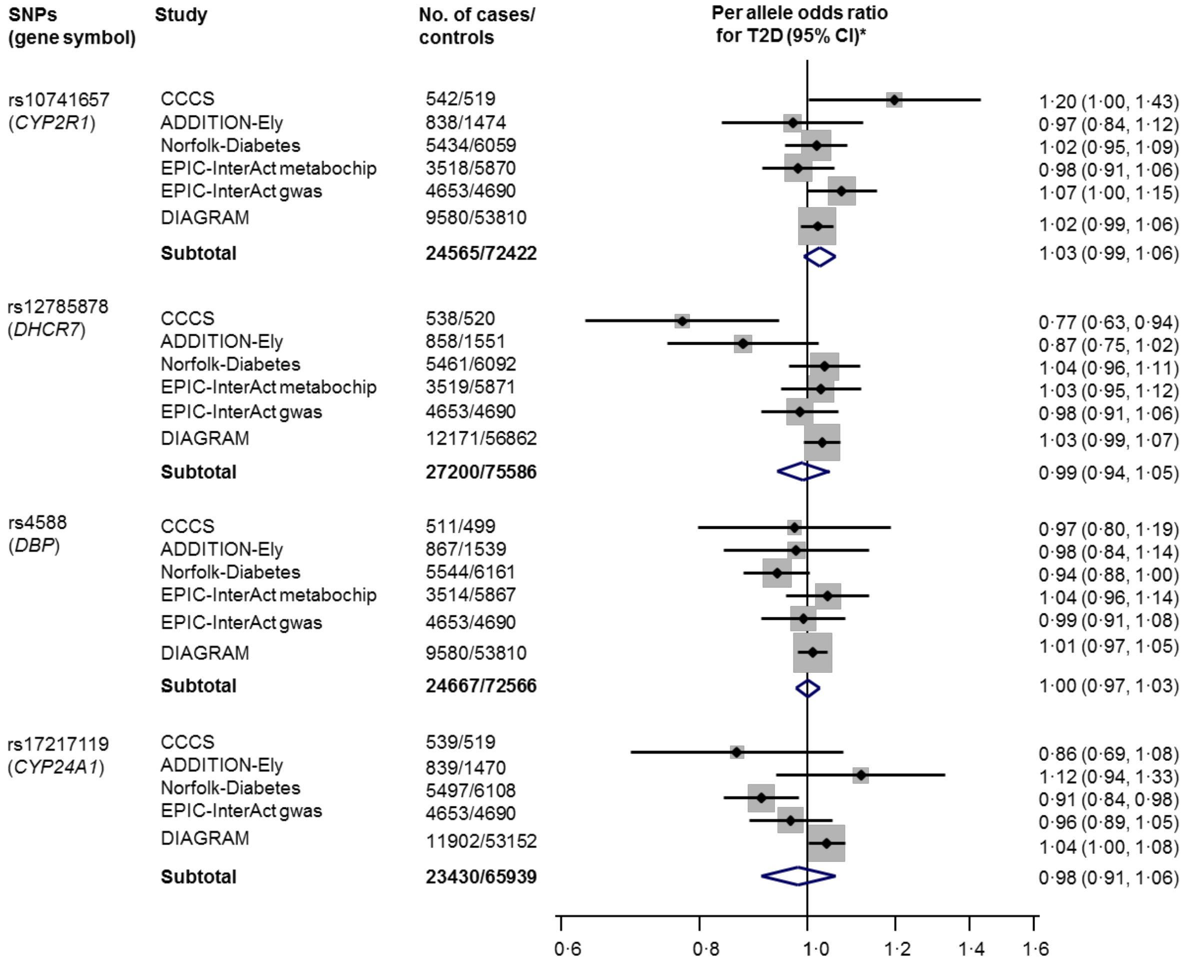

Supplementary Figure 3. Associations between four single nucleotide polymorphisms related to vitamin D metabolism and risk of type 2 diabetes.

* The allelic effect was fixed to the direction of 25(OH)D-lowering effects. For EPIC-InterAct study, the association between SNPs and T2D was estimated as hazard ratio. Abbreviations: 25(OH)D, 25-hydroxy vitamin D; SNP, single nucleotide polymorphism; CCCS, Cambridgeshire Case Control Study; CI, confidence interval; DIAGRAM, DIAbetes Genetics Replication And Meta-analysis; gwas, genome wide association study; T2D, type 2 diabetes;

Supplementary Table 4. Mendelian randomisation estimates of the association between genetically predicted concentrations of 25-hydroxy vitamin D and risk of type 2 diabetes.*

Gene symbol	Single nucleotide polymorphisms	Odds ratio	95% CI		*P*-value
Synthesis of 25(OH)D					
CYP2R1	rs10741657	1·21	0·93	1·57	0·15
DHCR7	rs12785878	0·90	0·51	1·58	0·71
CYP2R1, *DHCR7*	rs10741657; rs12785878	1·16	0·84	1·60	0·36
Metabolism of 25(OH)D					
DBP	rs4588	0·98	0·86	1·12	0·80
CYP24A1	rs17217119	0·82	0·40	1·72	0·61
DBP, *CYP24A1*	rs4588; rs17217119	0·95	0·59	1·52	0·82
CYP2R1, *DHCR7*, *DBP*, *CYP24A1*	4 SNPs*	1·01	0·75	1·36	0·94

*Results were based on multiple case-control and cohort analysis of 28,144 T2D cases and 76,344 non-cases (Supplementary Table 1 for details). The four estimates of the SNPs (*CYP2R1* rs10741657, *DHCR7* rs12785878, *DBP* rs4588, and *CYP24A1* rs17217119) were summarised into a single estimate by the likelihood-based mendelian randomisation analysis. Abbreviations: 25(OH)D, 25-hydroxy vitamin D; CI, confidence interval.

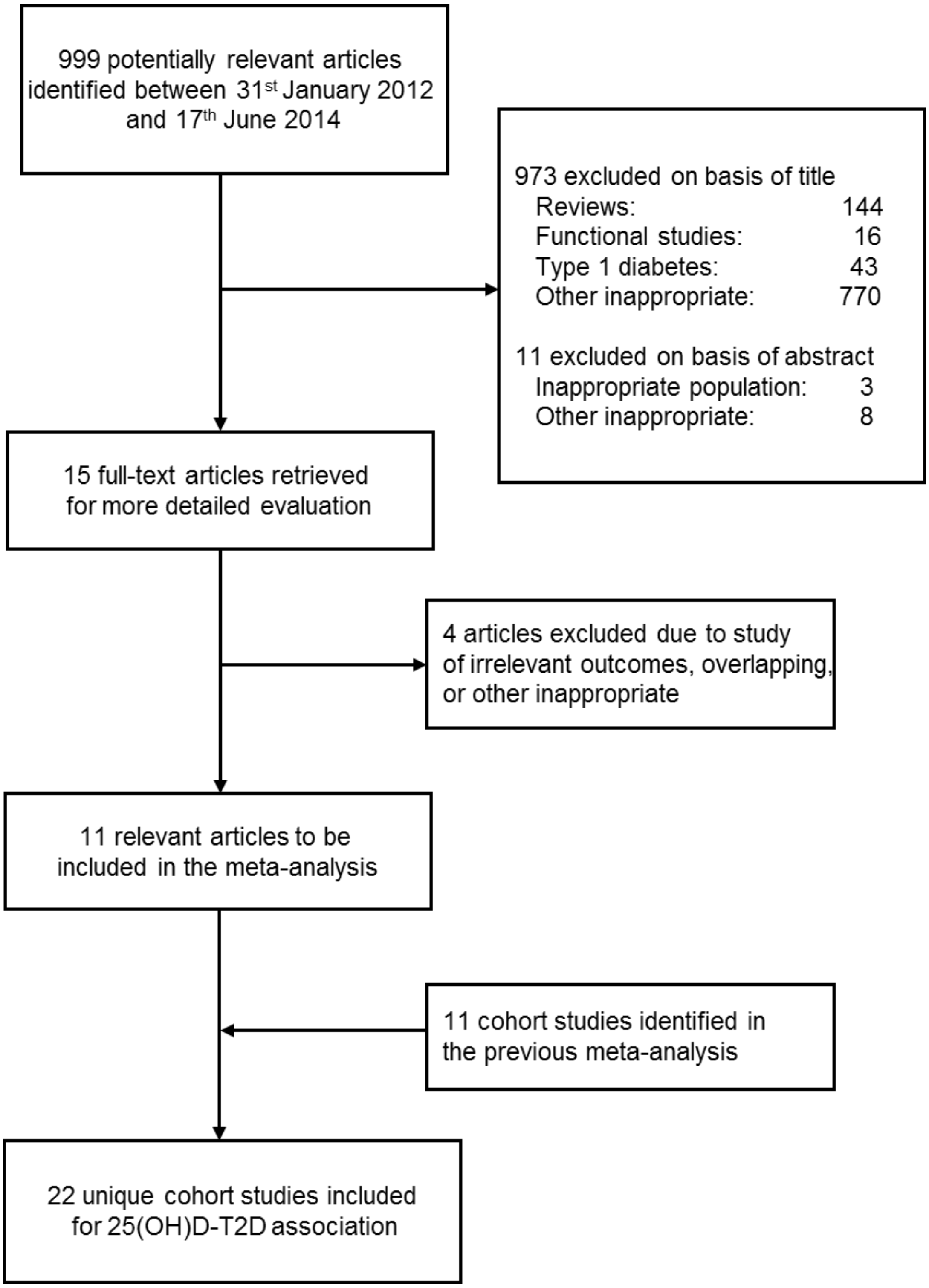

Supplementary Figure 4. Flow diagram of literature search and identification of studies for the association between circulating concentrations of 25-hydroxy vitamin D and risk of type 2 diabetes. Abbreviation: 25-hydroxy vitamin D: 25(OH)D; Type 2 diabetes: T2D.

Supplementary Table 5. Prospective studies of the association of circulating concentrations of 25-hydroxy vitamin D with risk of type 2 diabetes, newly included in the updated meta-analysis.*

Author, year, Study	Study design<br>Follow-up	Location	Demographics (age mean±SD or range, %women, ethnicity)	Sample size	Sample selection<br>Ascertainment of cases	Controls	25(OH)D assay;<br>Mean 25(OH)D (SD)	Adjustments
**Kayaniyil, S**, 2011, PROMISE[17]	Cohort<br>3 years of follow-up	Canada	50 ± 10 years<br>73% women<br>71% Caucasian	Cases: 30<br>Non cases: 489	OGTT	Non case cohort	Chemiluminescent immunoassay<br>58·0 (23·3) nmol/L	Age, sex, season, ethnicity, physical activity, change in physical activity, baseline vitamin D supplement use, change in vitamin D supplement use, BMI, and change in BMI
**Deleskog, A**, 2012, SDPP[18]	Nested case-control<br>8-10 years of follow-up	Sweden	35-56 years<br>Men / women<br>100% Caucasian	Cases: 279<br>Non cases: 1011	OGTT	Matched controls from cohort	Chemiluminescent immunoassay<br>61·4 (13·1) nmol/L	Age, sex, BMI, family history of diabetes, physical activity during leisure time, and BP
**Husemoen, LLN**, 2012, Inter99[19]	Cohort<br>5 years of follow-up	Demark	30-65 years<br>Men / women<br>100% Caucasian	Cases: 141<br>Non cases: 4155	OGTT, fasting glucose≥7 or 2h glucose≥11 mmol/L	Non case cohort	High-performance liquid chromatography<br>48·0 nmol/L	Season of blood collection, sex, age, family history of diabetes, BMI, change in weight during follow-up, leisure time physical activity, dietary habits, alcohol consumption, smoking status, total energy intake, social class, randomization group and self-reported changes in dietary habits, physical activity, and alcohol consumption during follow-up
**Hurskainen, AR**, 2012, KIHD[20]	Cohort<br>9 years of follow-up	Finland	62·9±6·5 years<br>Men / women<br>>99% European	Cases: 140<br>Non cases: 942	Fasting glucose≥6·1 mmol/L or OGTT 2-h glucose ≥10·0 mmol/L or treatment with insulin, oral, or dietary	Non case cohort	High-performance liquid chromatography<br>43·4 (17·6) nmol/L	Age, gender, examination year, BMI, waist-to-hip ratio, smoking, leisure-time physical activity intake of fruits, berries and vegetables, diabetes in family, and examination month
**Husemoen, LLN**, 2012, MONICA10[21]	Cohort<br>10 years of follow-up	Demark	55 (41-72) years<br>Men / women<br>100% Caucasian	Cases: 288<br>Non cases: 2283	Hospitalization with a diagnosis of diabetes (ICD-8: 249 or 250, ICD-10: DE10-14, DH36·0 or DO24 (excluding D=24·4); registration of chiropody (coded for diabetes) in the NHI service registry; frequent measurements of blood glucose either at least five times within a year or at least two annual measurements of glucose during a 5-year period; and prescription of insulin or oral anti-diabetic medication at least twice.	Non case cohort	Chemiluminescent immunoassay<br>61·2 nmol/L	Age, sex, season of blood collection, history of CVD, family history of diabetes; WC, physical activity during leisure time, healthy food index, fish intake, supplement use, smoking status, alcohol intake, and educational level

**Pilz, S**, 2012, Hoorn study[22]	Cohort<br>7·5 years of follow-up	Netherlands	67·9±5·7 years<br>51% women<br>100% Caucasian	Cases: 45<br>Non cases: 235	Fasting glucose≥7·0 mmol/L, 2h postload glucose ≥ 11·1 mmol/L or HbA1c ≥6·5% or glucose lowering drugs	Non case cohort	Radioimmunoassay<br>56·7 (18·8) nmol/L	Age, sex, BMI, fasting glucose, HDL-C, triglycerides, arterial hypertension, physical activity, season, parathyroid hormone, and GFR
**Pittas, AG**, 2012, DPP[23]	Cohort<br>2·7 years of follow-up	USA	51±11 years<br>67·2% women<br>57% Caucasian	Cases: 426<br>Non cases: 1613	OGTT	Non case with a vitamin D measure available	Liquid chromatography - tandem mass spectrometry<br>54·0 (24·3) nmol/L	Recruitment, age, sex, BMI, race, family history of diabetes, personal history of hypertension at baseline, smoking status at baseline, alcohol consumption, C-reactive protein, kidney function, self-reported physical activity, calcium intake, and treatment arm
**Afzal, S**, 2013, CCHS[24]	Cohort<br>29 years of follow-up	Demark	56 years<br>Men / women<br>100% Caucasian	Cases: 810<br>Non-cases: 9031	Self-reported diabetes and use of antidiabetic medicine at follow-up examination, nonfasting glucose >11 mmol/L at follow-up examination, or ICD8: 250 or ICD10: E11,13,14	Non case cohort	Chemiluminescent immunoassay<br>*Not available*	Age, sex, smoking status, duration and intensity of leisure time physical activity, income, and calendar month of blood draw
**Schottker, B**, 2013, ESTHER[25]	Cohort<br>8 years of follow-up	Germany	50-74 years<br>57·6% women<br>100% Caucasian	Cases: 829<br>Non-cases: 6962	GP'S medical records and HbA1c ≥6·5%	Non case cohort	Chemiluminescent immunoassay or liquid chromatography tandem-mass spectrometry<br>46·1 nmol/L (median)	Age, sex, season of blood draw, multi-vitamin supplements, frequent fish consumption, BMI, HbA1c, family history of diabetes, education, physical activity, smoking, hypertension, renal dysfunction, C-reactive protein, and fasting triglycerides
**Buijsse, B,** 2013, EPIC-Potsdam[26]	Case-cohort<br>6·6 years of follow –up	Germany	35-65 years<br>57% women<br>100% Caucasian	Cases: 1572<br>Non-cases: 2121	Self-reported diabetes and use of anti-diabetes medicine, or change in dietary behaviour due to diabetes	Non case cohort	Liquid chromatography - tandem mass spectrometry<br>47·1 nmol/L	Age, sex, centre, month of blood draw, education, smoking, alcohol intake, physical activity, BMI, waist circumference
**Schafer, AL,** 2014, OFS[27]	Cohort<br>8·6 years of follow-up	USA	≥65 years<br>100% women<br>100% Caucasian	Cases: 320<br>Non-cases: 5143	Self-reported and medication use	Non case cohort	Liquid chromatography - tandem mass spectrometry<br>57·4 (27·2) nmol/L	Age, clinic site, BMI, self-reported health and hypertension

* The current meta-analysis evaluated estimates from 22 studies. Eleven studies already evaluated in our prior meta-analysis[5] are not presented in the table.
Abbreviations: PROMISE, PROspective Metabolism and ISlet cell Evaluation; SDPP, Stockholm Diabetes Prevention Program; KIHD, Kuopio Ischaemic Heart Disease Risk Factor Study; MONICA, Danish Monitoring Trends and Determinants of Cardiovascular Disease; DPP, Diabetes Prevention Program; CCHS, Copenhagen City Heart Study; EPIC: European Prospective Investigation into Cancer and Nutrition; OFS: Osteoporotic Fractures Study; ICD-9, International Classification of Diseases 9th Revision; OGTT, oral glucose tolerance test.

References for online supplementary materials:

1. Pfister R, Sharp S, Luben R, et al. Mendelian randomization study of B-type natriuretic peptide and type 2 diabetes: evidence of causal association from population studies. *PLoS Med*. 2011;8(10):e1001112.
2. Williams DR, Wareham NJ, Brown DC, et al. Undiagnosed glucose intolerance in the community: the Isle of Ely Diabetes Project. *Diabet Med*. 1995;12(1):30–35.
3. Forouhi NG, Luan J, Hennings S, Wareham NJ. Incidence of Type 2 diabetes in England and its association with baseline impaired fasting glucose: the Ely study 1990-2000. *Diabet Med*. 2007;24(2):200–207.
4. Forouhi NG, Luan J, Cooper A, Boucher BJ, Wareham NJ. Baseline serum 25-hydroxy vitamin D is predictive of future glycemic status and insulin resistance: the Medical Research Council Ely Prospective Study 1990-2000. *Diabetes*. 2008;57(10):2619–25.
5. Forouhi NG, Ye Z, Rickard a P, et al. Circulating 25-hydroxyvitamin D concentration and the risk of type 2 diabetes: results from the European Prospective Investigation into Cancer (EPIC)-Norfolk cohort and updated meta-analysis of prospective studies. *Diabetologia*. 2012;55(8):2173–82.
6. Day N, Oakes S, Luben R, et al. EPIC-Norfolk: study design and characteristics of the cohort. European Prospective Investigation of Cancer. *Br J Cancer*. 1999;80 Suppl 1:95–103.
7. Halsall DJ, McFarlane I, Luan J, Cox TM, Wareham NJ. Typical type 2 diabetes mellitus and HFE gene mutations: a population-based case - control study. *Hum Mol Genet*. 2003;12(12):1361–1365.
8. Echouffo-Tcheugui JB, Simmons RK, Williams KM, et al. The ADDITION-Cambridge trial protocol: a cluster -- randomised controlled trial of screening for type 2 diabetes and intensive treatment for screen-detected patients. *BMC Public Health*. 2009;9:136.
9. Ye Z, Gillson C, Sims M, et al. The association of the mitochondrial DNA OriB variant (16184-16193 polycytosine tract) with type 2 diabetes in Europid populations. *Diabetologia*. 2013;56(9):1907–13.
10. The InterAct Consortium. Design and cohort description of the InterAct Project: an examination of the interaction of genetic and lifestyle factors on the incidence of type 2 diabetes in the EPIC Study. *Diabetologia*. 2011;54(9):2272–2282.
11. Morris AP, Voight BF, Teslovich TM, et al. Large-scale association analysis provides insights into the genetic architecture and pathophysiology of type 2 diabetes. *Nat Genet*. 2012;44(9):981–90.
12. Dupuis J, Langenberg C, Prokopenko I, et al. New genetic loci implicated in fasting glucose homeostasis and their impact on type 2 diabetes risk. *Nat Genet*. 2010;42(2):105–116.
13. Saxena R, Hivert M-F, Langenberg C, et al. Genetic variation in GIPR influences the glucose and insulin responses to an oral glucose challenge. *Nature Genet*. 2010;42(2):142–8.
14. Soranzo N, Sanna S, Wheeler E, et al. Common variants at 10 genomic loci influence hemoglobin A1(C) levels via glycemic and nonglycemic pathways. *Diabetes*. 2010;59(12):3229–39.
15. DerSimonian R, Laird N. Meta-analysis in clinical trials. *Control Clin Trials*. 1986;7(3):177–188.
16. Higgins JPT, Thompson SG, Deeks JJ, Altman DG. Measuring inconsistency in meta-analyses. *BMJ*. 2003;327(7414):557–560.
17. Kayaniyil S, Retnakaran R, Harris SB, et al. Prospective associations of vitamin D with beta-cell function and glycemia: the PROspective Metabolism and ISlet cell Evaluation (PROMISE) cohort study. *Diabetes*. 2011;60(11):2947–2953.
18. Deleskog A, Hilding A, Brismar K, Hamsten A, Efendic S, Ostenson CG. Low serum 25-hydroxyvitamin D level predicts progression to type 2 diabetes in individuals with prediabetes but not with normal glucose tolerance. *Diabetologia*. 2012;55(6):1668–1678.
19. Husemoen LL, Thuesen BH, Fenger M, et al. Serum 25(OH)D and type 2 diabetes association in a general population: a prospective study. *Diabetes Care*. 2012;35(8):1695–1700.
20. Hurskainen AR, Virtanen JK, Tuomainen TP, Nurmi T, Voutilainen S. Association of serum 25-hydroxyvitamin D with type 2 diabetes and markers of insulin resistance in a general older population in Finland. *Diabetes Metab Res Rev*. 2012;28(5):418–423.
21. Husemoen LL, Skaaby T, Thuesen BH, Jorgensen T, Fenger R V, Linneberg A. Serum 25(OH)D and incident type 2 diabetes: a cohort study. *Eur J Clin Nutr*. 2012;66(12):1309–1314.
22. Pilz S, van den Hurk K, Nijpels G, et al. Vitamin D status, incident diabetes and prospective changes in glucose metabolism in older subjects: the Hoorn study. *Nutr Metab Cardiovasc Dis*. 2012;22(10):883–889.
23. Pittas AG, Nelson J, Mitri J, et al. Plasma 25-hydroxyvitamin D and progression to diabetes in patients at risk for diabetes: an ancillary analysis in the Diabetes Prevention Program. *Diabetes Care*. 2012;35(3):565–573.
24. Afzal S, Bojesen SE, Nordestgaard BG. Low 25-hydroxyvitamin D and risk of type 2 diabetes: a prospective cohort study and metaanalysis. *Clin Chem*. 2013;59(2):381–391.

25. Schottker B, Herder C, Rothenbacher D, Perna L, Muller H, Brenner H. Serum 25-hydroxyvitamin D levels and incident diabetes mellitus type 2: a competing risk analysis in a large population-based cohort of older adults. *Eur J Epidemiol*. 2013;28(3):267–275.
26. Buijsse B, Boeing H, Hirche F, et al. Plasma 25-hydroxyvitamin D and its genetic determinants in relation to incident type 2 diabetes: a prospective case-cohort study. *Euro J Epidemiol*. 2013;28(9):743–52.
27. Schafer AL, Napoli N, Lui L, Schwartz A V, Black DM. Serum 25-hydroxyvitamin D concentration does not independently predict incident diabetes in older women. *Diab Med*. 2014;31(5):564–9.
